# Mobile Robot Self-Localization System Using Single Webcam Distance Measurement Technology in Indoor Environments

**DOI:** 10.3390/s140202089

**Published:** 2014-01-27

**Authors:** I-Hsum Li, Ming-Chang Chen, Wei-Yen Wang, Shun-Feng Su, To-Wen Lai

**Affiliations:** 1 Department of Information Technology in Lee-Ming Institute of Technology, No.2-2, Lizhuan Rd., Taishan Dist., New Taipei 243, Taiwan; E-Mail: i-hsum@orion.ee.ntust.edu.tw; 2 Department of Electrical Engineering, National Taiwan University of Science and Technology, 43 Keelung Road, Section 4, Taipei 106, Taiwan; E-Mails: maximchen.chen@gmail.com (M.-C.C.); sfsu@mail.ntust.edu.tw (S.-F.S.); 3 Department of Applied Electronics Technology, National Taiwan Normal University, 160, He-ping East Rd., Section 1, Taipei 106, Taiwan; E-Mail: blanc131@gmail.com

**Keywords:** indoor robot localization, image-based distance measurement system, parallel lines distance measurement system

## Abstract

A single-webcam distance measurement technique for indoor robot localization is proposed in this paper. The proposed localization technique uses webcams that are available in an existing surveillance environment. The developed image-based distance measurement system (IBDMS) and parallel lines distance measurement system (PLDMS) have two merits. Firstly, only one webcam is required for estimating the distance. Secondly, the set-up of IBDMS and PLDMS is easy, which only one known-dimension rectangle pattern is needed, *i.e.*, a ground tile. Some common and simple image processing techniques, *i.e.*, background subtraction are used to capture the robot in real time. Thus, for the purposes of indoor robot localization, the proposed method does not need to use expensive high-resolution webcams and complicated pattern recognition methods but just few simple estimating formulas. From the experimental results, the proposed robot localization method is reliable and effective in an indoor environment.

## 1. Introduction

Autonomous robots have a wide range of potential applications in security guards, house cleaning and even warfare. Most of them are equipped with position measurement systems (PMSs) for the purpose of precisely locating themselves and navigating in their working fields. Three typical techniques [1] in PMSs are triangulation, scene analysis, and proximity. The triangulation technique uses the geometric properties of triangles to compute object locations. The most well-known technique is the Global Positioning System (GPS). However, GPS, as it is satellite dependent, has an inherent problem of accurately determining the locations of objects within a building [2]. A proximity location-sensing technique entails determining when an object is “near” a known location, and the object's presence can be sensed via some limited range physical phenomenon. Some famous techniques are detecting physical contact [3,4] or monitoring wireless cellular access points [5,6]. The scene analysis location sensing technique uses features of a scene observed from a particular vantage point to draw conclusions about the location of the observer or of objects in the scene. Some well-known techniques are a radar location system [7] or a visual images location system [8].

In an indoor localization technique, the infrared light [6], ultrasonic [9], laser range finder [10,11], RFID [12], and radar [13] are the most popular wireless techniques. Diffuse infrared technology is commonly used to realize indoor locations, but the short-range signal transmission and line-of-sight requirements limit the growth. Ultrasonic localization [9] uses the time-of-flight measurement technique to provide location information. However, the use of ultrasound requires a great deal of infrastructure in order for it to be highly effective and accurate. Laser distance measurement is executed by measuring the time that it takes for a laser light to be reflected off a target and returned back to the sender. Because the laser range finder is a very accurate and quick measurement device, this device is widely used in many applications. In [10,11], Subramanian *et al.* and Barawid *et al.* proposed an autonomous vehicle guidance system based on a laser rangefinder. The laser rangefinder was used to acquire environment distance information that can be used to identify and avoid obstacles during navigation. In [14], Thrun *et al.* provided an autonomous navigation method based on a particle filter algorithm. In this study, the laser rangefinder can receive all the measurement information that it can utilize to compute the likelihood of the particles. These papers confirm that laser rangefinders are high performance and high accuracy measurement equipment. However, their high performance relies on high hardware costs. RFID-based localization uses RF tags and a reader with an antenna to locate objects, but the detection of each tag only can work over approximately 4 to 6 meter distances. To improve the low precision on location positioning, the well-known SpotON [15] technology uses an aggregation algorithm based on radio signal strength analysis for 3D-location sensing. However, a complete system is not available yet. An RF-based RADAR system [7,16,17] uses the 802.11 network adapter to measure signal strengths at multiple base stations positioned to provide overlapping coverage for locating and tracking objects inside buildings. Unfortunately, most cases to date cannot provide overall accuracy of systems as optimal as desired. In indoor localization for robots, most of these wireless techniques are used to perform scans of static obstacles around the robots, and the localization is calculated by matching those scans with a metric map of the environment [18,19], but in dynamic environments the detected static-features are often not enough for estimating a robust localization.

Li *et al.* [20] proposed a NN-based mobile phone localization technique using Bluetooth connectivity. In this large-scale network, mobile phones equipped with GPS represent beacons, and others could connect to the beacon phones with Bluetooth connectivity. By formulating the Bluetooth network as an optimization problem, a recurrent neural network is developed to distributively find the solutions in real time. However, in general, the sampling rate of Bluetooth is relatively low, and then accurately estimating a moving object in real time is not easy. In [21], a recurrent neural network was proposed to search a desirable solution for a range-free localization of WSNs under the condition that the WSNs can be formed as a class of nonlinear inequalities defined on a graph. Taking advantage of parallel computation of the NN, the proposed approach can effectively solve the WSN localization problem, although the limited transmission bandwidth might cause difficulty in the localization.

Recently, image-based techniques have been preferred over wireless techniques [4,5,9,22]; this is because they are passive sensors and are not easily disturbed by other sensors. In [1], a portable-PC capable of marker detection, image sequence matching, and location recognition was proposed for an indoor navigation task. JongBae *et al.* used the augmented reality (AR) technique to achieve an average location recognition success rate of 89%, though the extra cost must be considered in this technique. In [23], Cheoket *et al.* provided a method of localization and navigation in wide indoor areas with a wearable computer for human-beings. Though the set-up cost is lower, this method is not easy to implement and set up if users do not know the basic concept of electronic circuit analysis and design. Furthermore, an imaged-based method for distance measurement was proposed in [24–29]. According to the transform equations in those papers, the distance can be calculated from the ratio of the size between the pre-defined reference points and the measured object. In recent years, we have seen growing importance placed on research in two-camera localization systems [30,31]. From two different images, the object distances can be calculated by a triangular relationship. However, to ensure the measuring reliability, the photography angle and the distance between two cameras must be maintained at the same position. Due to the use of two cameras for the measuring device, the set-up costs of the experimental environment will be increased.

Nowadays, surveillance systems exist in most modern buildings, and cameras have been configured around these buildings. In general, one camera covers one specific area. In order to locate an autonomous patrolling robot using existing cameras in buildings, a single-camera localization technique must be developed for the patrolling robots. This study aims to develop a single-webcam distance measurement technique for indoor robot localization with the purposes of saving set-up costs and increasing the accuracy of distance measurements. In our approach, the working area setting can be as simplified as possible, because the existing webcams in the surveillance environment can be utilized without any change. For a single webcam in its working coverage area, we develop an improved image-based distance measurement system (IBDMS) and a parallel lines distance measurement system (PLDMS) to measure the location of a robot according to a known-size rectangle pattern, *i.e.*, a ground tile. This measurement system uses four points, *i.e.*, the four corners of a ground tile, to form a pair of parallel lines in the webcam image. Referring to the pair of parallel lines, we can measure the location of a robot within the visual range of a webcam. Because of the fixed monitoring area of an individual webcam, few simple image processing strategies are used to search for the robots before going through IBDMS and PLDMS. First, we use the low-pass filter and on-line background update method to reduce background noise, and adopt the image morphology to complete prospect information and to remove the slight noise. When the mobile robot is located, IBDMS and PLDMS can obtain the real-world coordinates of a mobile robot. Finally, the localization of a mobile robot can be shown on the two-dimensional map immediately. Thus, for the purpose of indoor robot localization, the proposed method does not need to use complicate pattern recognition methods, but just few simple estimation formulas.

## 2. Photography Methods

Before locating a robot by the proposed single-webcam localization technique, the acquired images must go through photograph processing for removing noise and unnecessary information. These techniques include a gray scale, a background subtraction, a morphological image processing, and a connected components labeling technique. Next, we briefly discuss the procedures [32] of these photographic correction techniques used in this paper.

### 2.1. Camera Calibration

Distortion could happen in captured images, especially is cheap webcams are used. To attenuate distortion of the captured images and thus increase the accuracy of the robot location task, the camera calibration should be done before the localization is attempted. OpenCV has taken into account the radial and tangential factors for the image distortion problem. The radial factor can be calculated by the following equations:

(1) $$x_{\text{corrected}}=x(1+k_{1}r^{2}+k_{2}r^{4}+k_{3}r^{6})$$

(2) $$y_{\text{corrected}}=y(1+k_{1}r^{2}+k_{2}r^{4}+k_{3}r^{6})$$

The tangential distortion can be corrected via the equations as follows:

(3) $$x_{\text{corrected}}=x+[2p_{1}xy+p_{2}(r^{2}+2x^{2})]$$

(4) $$y_{\text{corrected}}=y+[p_{1}(r^{2}+2y^{2})+2p_{2}xy]$$

In Equations (1–4) the pixel (*x*,*y*) is the image coordinate in the input image and (*x_corrected_, y_corrected_*) is the image coordinate in the corrected output image. The distortion coefficient vector can be represented as *c_di_*=[*k*_1_ *k*_2_ *p*_1_ *p*_2_ *k_3_*] Moreover, the unit conversion can be represented as:

(5) $$\left[\begin{array}{c} x\\ y\\ w \end{array}\right]=\left[\begin{array}{ccc} f_{x} & 0 & c_{x}\\ 0 & f_{y} & c_{y}\\ 0 & 0 & 1 \end{array}\right]\;\left[\begin{array}{c} x_{\text{before}}\\ y_{\text{before}}\\ z_{\text{before}} \end{array}\right]$$

**Figure 1.**
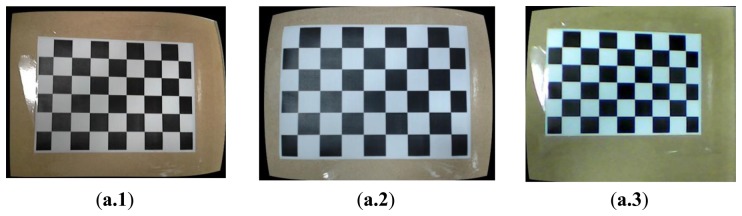

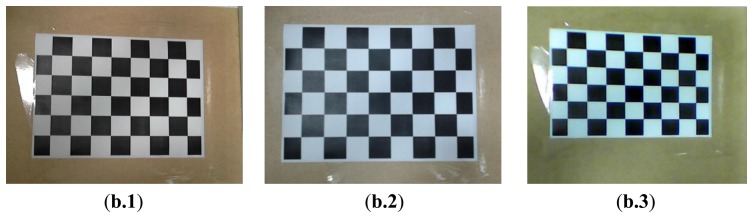
Calibration procedure for the webcam images. (**a**) Before the calibration produces for three webcams, (**a.1**) Logitech HD Webcam C310; (**a.2**) Logitech HD Pro Webcam C920; (**a.3**) Ronald HD Webcam PC235. (**b**) Images after the calibration produces for three webcams, (**b.1**) Logitech HD Webcam C310; (**b.2**) Logitech HD Pro Webcam C920; (**b.3**) Ronald HD Webcam PC235.

Where *w* is explained by the use of homography coordinate system (and *w*=*z_before_*), *f_x_* and *f_y_* are the camera focal length, and *c_x_* and *c_y_* are the optical centers expressed in pixels coordinates. After calculating the camera Equation (5) and the distortion coefficient *c_di_*, the functions *initUndistortRectifyMap*() and the *remap*() can calibrate the distorted images. Figure 1a shows the images before the calibration done for three webcams, and Figure 1b shows the images after the calibration procedure. For the 1st webcam (HD Webcam C310, Logitech, Lausanne, Switzerland) in our experimental environment, the distortion coefficient vector is:

(6) $$c_{d1}=[\begin{array}{ccccc} 0.0895 & ‐0.7064 & ‐0.0034 & 0.0020 & 1.3227 \end{array}]$$

and the camera conversion matrix is:

(7) $$\left[\begin{array}{c} x\\ y\\ w \end{array}\right]=\left[\begin{array}{ccc} 518.38 & 0 & 253.56\\ 0 & 518.07 & 644.23\\ 0 & 0 & 1 \end{array}\right]\;\left[\begin{array}{c} x_{\text{before}}\\ y_{\text{before}}\\ z_{\text{before}} \end{array}\right]$$

For the 2nd webcam (HD Pro Webcam C920, Logitech, Lausanne, Switzerland) in our experimental environment, the distortion coefficient vector is:

(8) $$c_{d2}=[\begin{array}{ccccc} 0.2460 & ‐1.8737 & ‐0.0023 & ‐0.0043 & 5.4119 \end{array}]$$

and the camera conversion matrix is:

(9) $$\left[\begin{array}{c} x\\ y\\ w \end{array}\right]=\left[\begin{array}{ccc} 598.36 & 0 & 318\\ 0 & 600.76 & 264.19\\ 0 & 0 & 1 \end{array}\right]\;\left[\begin{array}{c} x_{\text{before}}\\ y_{\text{before}}\\ z_{\text{before}} \end{array}\right]$$

For the 3rd webcam (HD Webcam PC235, Ronald, Osaka, Japan) in our experimental environment, the distortion coefficient vector is:

(10) $$c_{d3}=[\begin{array}{ccccc} 0.8433‐ & 12.8097 & ‐0.0339 & ‐0.0185 & 111.8152 \end{array}]$$

and the camera conversion matrix is:

(11) $$\left[\begin{array}{c} x\\ y\\ w \end{array}\right]=\left[\begin{array}{ccc} 1272.37 & 0 & 378.46\\ 0 & 1283.87 & 249.52\\ 0 & 0 & 1 \end{array}\right]\;\left[\begin{array}{c} x_{\text{before}}\\ y_{\text{before}}\\ z_{\text{before}} \end{array}\right]$$

### 2.2. Image Segmentation

In a grayscale image, the value of each pixel carries only intensity information. It is known as a black-and-white image, which is composed exclusively of shades of gray. Black is at the weakest intensity and white is at the strongest one. The gray scale technique can change a color image into a black-and-white image. The luminance *f*_1_(*x*, *y*) of the *pixel*(*x*, *y*) is described as:

(12) $$f_{1}(x,y)=0.299R_{\left(x,y\right)}+0.587G_{\left(x,y\right)}+0.114B_{\left(x,y\right)}$$

where *R_(x,y)_*, *G_(x,y)_*, and *B_(x,y)_* are color values at the *pixel*(*x*,*y*). Equation (12) can create binary images by a threshold value *t* from a grayscale image:

(13) $$f_{\text{binary}}(x,y)=\{\begin{array}{ccc} 255 & if & f_{1}(x,y)\geq t\\ 0 & \text{else} & \end{array}$$

Subtracting a binary acquired image *f_current_*(*x*, *y*) from a binary background image *f_bg_*(*x*, *y*), we can obtain a binary foreground image as:

(14) $$f_{fg}(x,y)=\left|f_{bg}(x,y)-f_{\text{current}}(x,y)\right|$$

### 2.3. Morphological Image Processing

After the process of image segmentation, discontinuous edges and noise may happen in a foreground image. These will cause wrong judgments during object identification. Therefore, this paper utilizes some morphological image processing operations, such as dilation, erosion, opening and closing, in order to enable the underlying shapes to be identified and optimally reconstruct the image from their noisy precursors.

### 2.4. Connected-Components Labeling

The aim of connected-component labeling is to identify connected-components that share similar pixel intensity values, and then to connect them with each other. The connected-component labeling scans an image and groups pixels into one or more components according to pixel connectivity. Once all groups are determined, each pixel is labeled with a grey level on the basis of the component.

**Figure 2.**
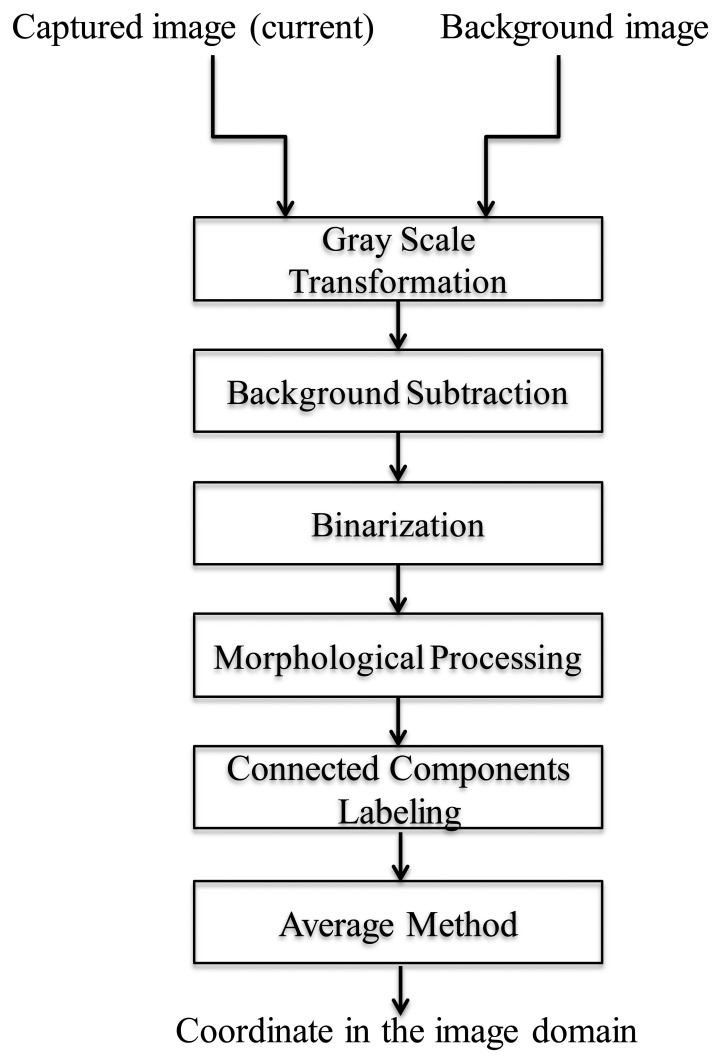
Procedures of the image processing techniques for the robot localization.

**Figure 3.**
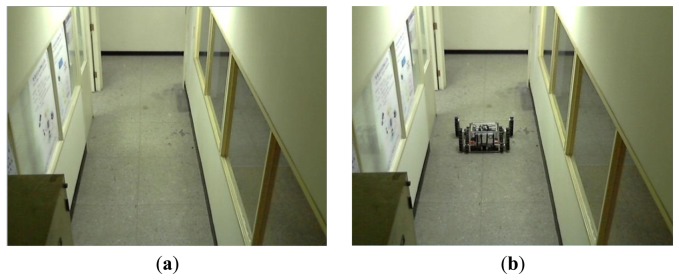

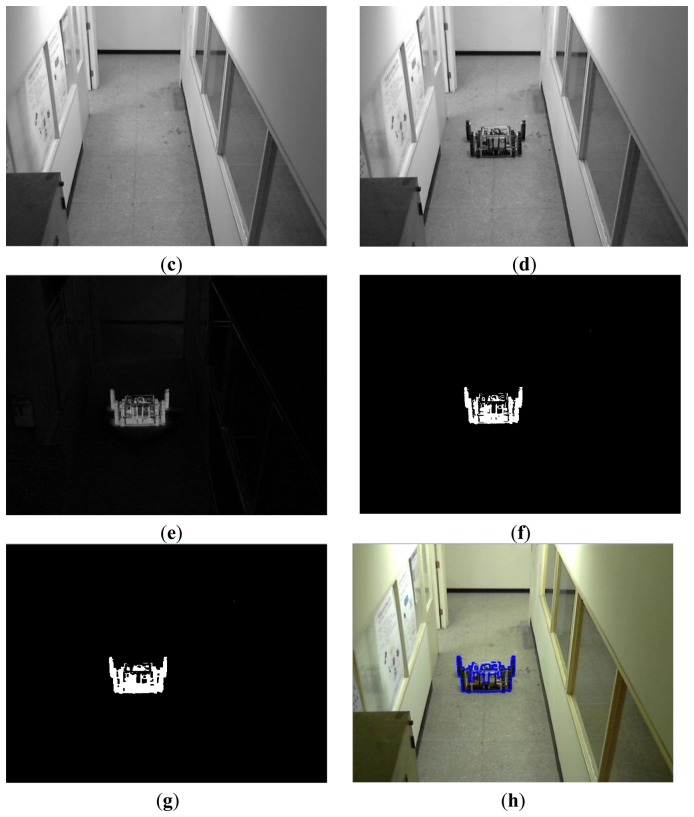
(**a**) Background image. (**b**) Captured image. (**c**) Gray scale of the background image. (**d**) Gray scale of the captured image. (**e**) Background subtraction. (**f**) Binarization processing of the foreground image. (**g**) Morphological processing of the foreground image. (**h**) Connected components labeling.

**Figure 4.**
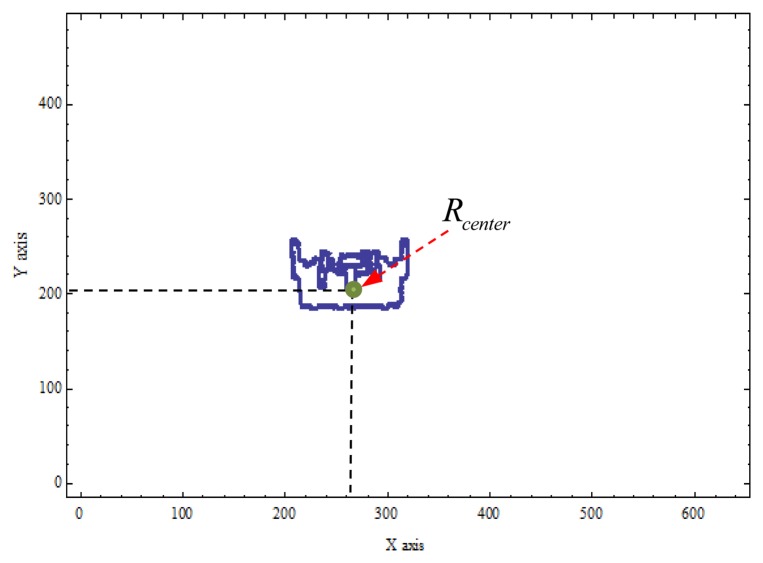
Coordinates of the mobile robot in the image-domain.

According to the aforementioned discussions, we can locate a robot in the captured image in an image-domain. Figure 2 shows the overall schemes of the image processing, and the experimental results are shown in Figure 3. In Figure 4, *R_center_* is the center of the robot in the processed image and can be easily calculated by the simple average method. In this paper, *R_center_* stands for the center-coordinate of the robot in the image-domain.

## 3. Mobile Robot Localization System with Single Webcam

After the captured images go through image processing, we can locate the robot in the image-domain. Then, we should calculate the coordinates of the robot in the image. That is, two distances, the x-axis and the y-axis, should be determined: 1. *d_i_* represents the distance between *R_center_* and the webcam: 2. *w_i_* represents the distance between *R_center_* and the wall, as shown in Figure 4. In this paper the IBDMS is used to calculate the distance *d_i_*, and the PLDMS is used to calculate the distance *w_i_*.

### 3.1. Experimental Map

**Figure 5.**
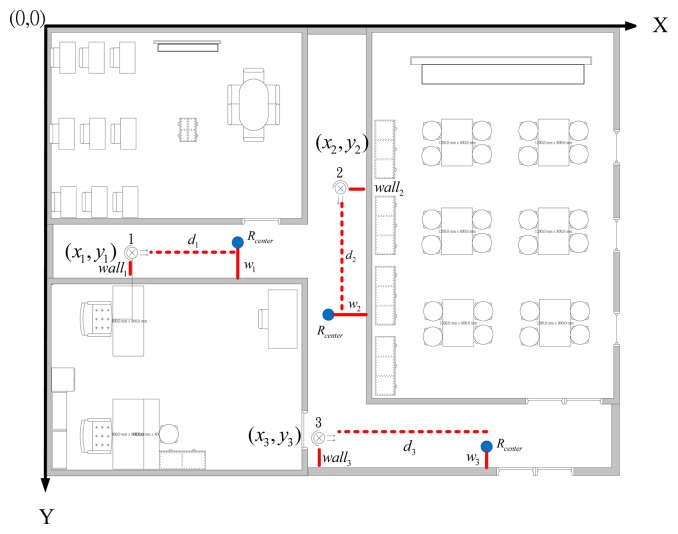
Map of our experimental location.

Figure 5 shows the map of our experimental environment. In the map, the coordinate of the first webcam is set to (*x*_1_, *y*_2_), the second one is set to (*x*_2_, *y*_2_), and the third one is set to. (*x*_3_, *y*_3_) *wall_i_* (*i*=1,2,3) represents the distance between the *i*th webcam and the wall. *d_i_* (*i*=1,2,3) represents the distance between the *i*th webcam and *R_center._* *w_i_* (*i*=1,2,3) is the distance between the wall and *R_center._* in the *i*th webcam covering area. Clearly, the coordinates of webcams (*x_i_,y_i_*) and the distances *wall_i_* are given. Therefore, if distances *d_i_* and *w_i_* can be calculated, we can easily represent the robot with its coordinate in the area, which is covered by one of the set-up webcams. The equations for calculating the coordinate of the robot are defined in Equations (15–17):

(15) $${\text{webcama}}_{1}:\{\begin{array}{l} X_{1}=x_{1}+d_{1}\\ Y_{1}=y_{1}+({\text{wall}}_{1}-w_{1}) \end{array}$$

(16) $${\text{webcama}}_{2}:\{\begin{array}{l} X_{2}=x_{2}-({\text{wall}}_{2}-w_{2})\\ Y_{2}=y_{2}+d_{2} \end{array}$$

and:

(17) $${\text{webcama}}_{3}:\{\begin{array}{l} X_{3}=x_{3}+d_{3}\\ Y_{3}=y_{3}+({\text{wall}}_{3}-w_{3}) \end{array}$$

Where (*x_i_,y_i_*) (*i*=1,2,3) are the coordinate of the robot in the covering area of the *i*th webcam.

### 3.2. Calculation of Distance d_i_ with IBDMS [24–29]

**Figure 6.**
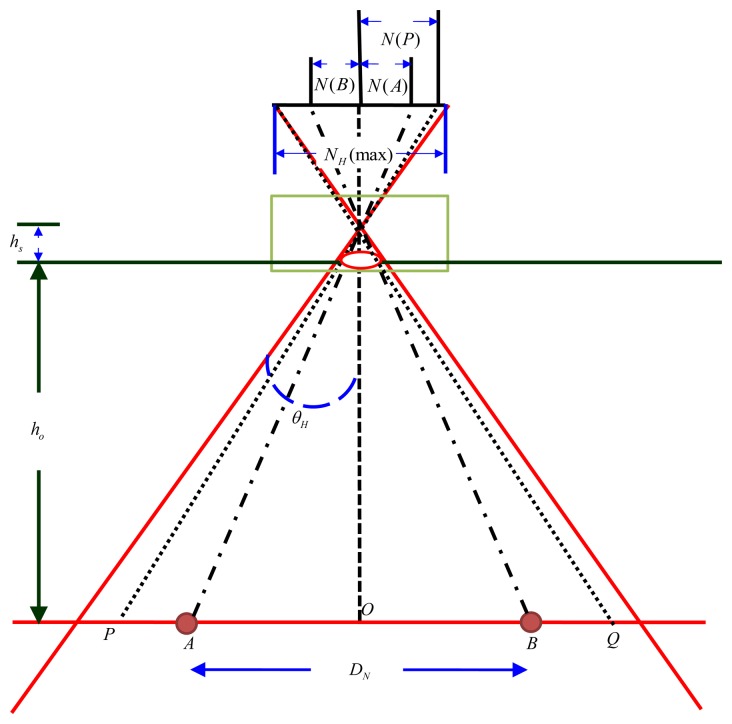
Schematic diagram of the IBDMS.

IBDMS is developed in this paper for the purpose of calculating the distances *d_i_* (*i*=1,2,3), which can work on a single webcam and only depends on a known-dimension rectangle, *i.e.*, a ground tile. The idea of IBDMS is from the triangular relationship, shown in Figure 6, that is we first capture an image incorporating a known-dimension rectangle, and then the proportion relationship between the real-dimension and the image-dimension of the rectangle can be found. According to the proportion relationship, the distance *d_i_* can then be easily calculated. Figure 6 shows the IBDMS set-up. It only requires a webcam and two given-location points *A* and *B*, which could be two corners of a ground tile. *h_s_* is a constant parameter of the webcam *O* is the intersection of the optical axis and the plane. The targeted objects which lie on the plane and are perpendicular to the optical axis can be measured by simple trigonometric function derivations. Hence, *h_o_* can expressed as:

(18) $$h_{o}=\frac{D_{N}}{N(A)+N(B)}N_{H\max}\text{Cot}\theta_{H}-h_{s}$$

where *h_o_* can be considered as any of distances *d_i_* (*i*=1, 2, 3) for the *i*th webcam, *N*(*A*) and *N*(*B*) are pixel values in the captured image *N_H_*(max) is the maximal pixel width in a horizontal scan line of the image. *D_N_* is the width between points *A* and *B. θ_H_* is the horizontal view angle.

### 3.3. Calculation of Distance w_i_ with PLDMS

**Figure 7.**
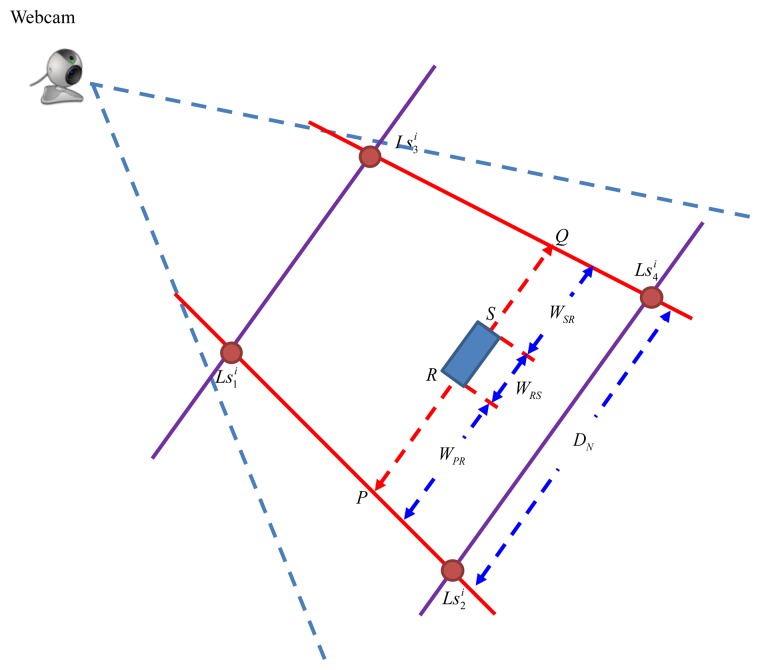
Diagram of the PLDMS.

Inheriting the concept of the IBDMS, a parallel-line distance measurement system (PLDMS) for measuring the distances *w_i_* is developed. Figure 7 shows the schematic diagram of the PLDMS. In PLDMS, four points, $Ls_{1}^{i}$, $Ls_{2}^{i}$, $Ls_{3}^{i}$, and $Ls_{4}^{i}$, are considered as the reference points. In Figure 7, we draw a pair of parallel lines ( $\bar{Ls_{1}^{i}Ls_{2}^{i}}$ and $\bar{Ls_{3}^{i}Ls_{4}^{i}}$) through these points *D_N_* is the width between $\bar{Ls_{1}^{i}Ls_{2}^{i}}$ and $\bar{Ls_{3}^{i}Ls_{4}^{i}}$. Furthermore, in the image-domain, as Figure 7, the linear proportion of the line ( $\bar{Ls_{3}^{i}Ls_{4}^{i}}$) and the line ( $\bar{Ls_{1}^{i}Ls_{2}^{i}}$) can be defined as:

(19) $$\bar{Ls_{1}^{i}Ls_{2}^{i}:}\frac{y^{i}-y_{1}^{i}}{y_{2}^{i}-y_{1}^{i}}=\frac{x^{i}-x_{1}^{i}}{x_{2}^{i}-x_{1}^{i}}$$

(20) $$\bar{Ls_{3}^{i}Ls_{4}^{i}:}\frac{y^{i}-y_{3}^{i}}{y_{4}^{i}-y_{3}^{i}}=\frac{x^{i}-x_{3}^{i}}{x_{4}^{i}-x_{3}^{i}}$$

and:

(21) $$N_{H}(PQ)=\bar{Ls_{1}^{i}Ls_{2}^{i}}{\left(y^{i}\right)}_{l_{PQ}}-\bar{Ls_{3}^{i}Ls_{4}^{i}}{\left(y^{i}\right)}_{l_{PQ}}$$

where ( $x_{j}^{1}$, $y_{j}^{i}$) (*j*=1,2,3,4)are, respectively, the image coordinate of the points $Ls_{j}^{i}$, and the pixel (*x^i^*, *y^i^*)(*i*=1,2,3) is any image points laying at the line $\bar{Ls_{1}^{i}Ls_{2}^{i}}$ or $\bar{Ls_{3}^{i}Ls_{4}^{i}}$. In Figure 7, *N_H_*(*PQ*) is the number of pixels between *P* and *Q*, which can be obtained by Equation (21).

**Figure 8.**
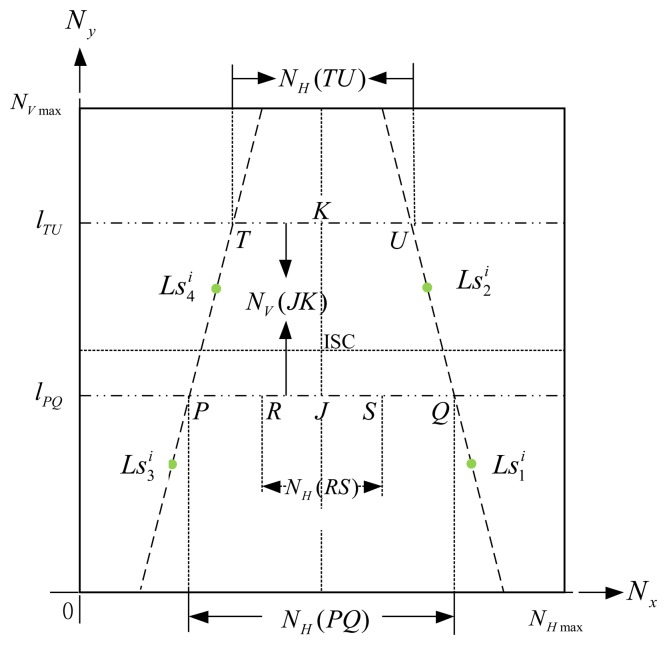
Schematic diagram of the PLDMS.

In Figure 8, points *_R_* and *S* could be two corners of a known-dimension rectangle, *i.e.*, a ground tile, in a captured image (image-domain), and *P* and *Q* are the cross points between the scan line *l_PQ_* and the lines $\bar{Ls_{1}^{i}Ls_{2}^{i}}$ and $\bar{Ls_{3}^{i}Ls_{4}^{i}}$. For the reason that *P*, *Q*, *R*, and *S* are laying at the same scan line, we can easily calculate the points *_P_* and *Q* by Equations (19) and (20), where *P*, *S* and, ( $x_{j}^{i}$, $y_{j}^{i}$) (*j*=1,2,3,4) have been given by the image processes. In our experiments, the width between *R* and *S* means the width of the robot. Furthermore, the width *W_RS_* can be calculated by the proportion relationship, which is defined as:

(22) $$W_{RS}=D_{N}\times \frac{N_{H}(RS)}{N_{H}(PQ)}$$

where *N_H_*(*RS*) and *N_H_*(*PQ*) can be obtained by the PLDMS. With the same idea, we can calculate the width *W_PR_* or the width *W_SQ_*.

### 3.4. Overall Procedures of the Proposed Localization Method

**Figure 9.**
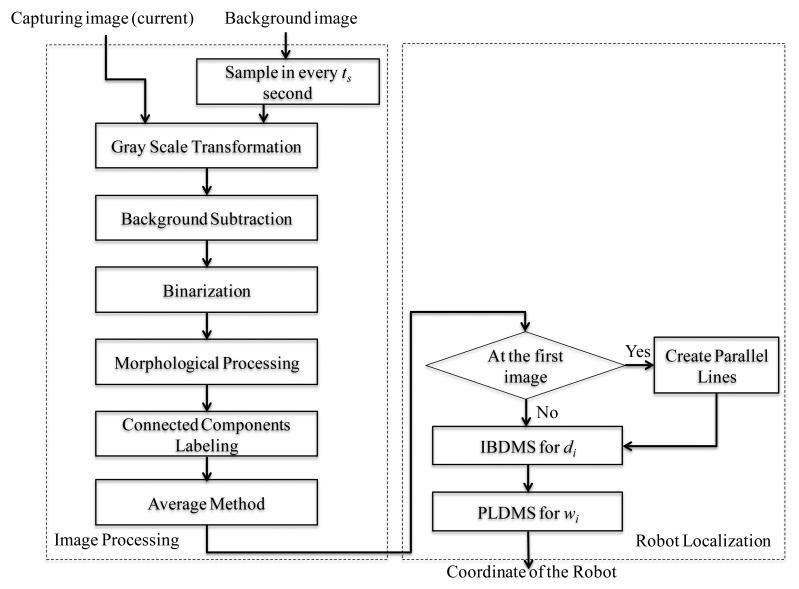
Overall scheme of the proposed localization method.

**Figure 10.**
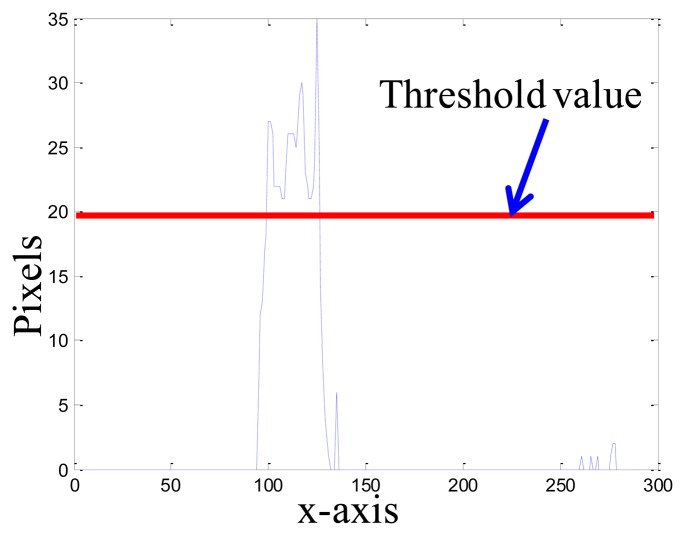
Update of the background image by using segmentation horizontal projection.

Figure 9 shows the overall scheme of the proposed robot localization method, where *t_s_* is the update time-interval for the background image. The moment of updating the background image is that if the segmentation horizontal projection of the binary foreground image *f_fg_*(*x, y*) is bigger than the threshold value *t*_1_, the captured image *f_current_*(*x, y*) is set to a new background image; otherwise, we keep the original background image. In Equation (23), the function *s*• means the process of the segmentation horizontal projection, as shown in Figure 10.

(23) $$f_{bg}(x,y)=\{\begin{array}{ccc} f_{\text{current}} & if & s(f_{fg}(x,y))<t_{1}\\ f_{\text{current}} & \left(x,y\right) & \text{otherwise} \end{array}$$

In the image processing, the coordinates of the robot can be calculated through the methods of gray-scale transformation, background subtraction, binarization, morphological processing, connected components labeling, and averaging method. The background image is updated in every *t_s_* seconds with the update Equation (23). After image processing, the coordinate in the image-domain goes through the IBDMS for calculating the distance *d_s_* (Equation (18)) and the PLDMS for calculating the width *w_i_* (Equation (22)). Then, we can locate the robot in the real-world domain.

## 4. Experimental Results

### 4.1. Set-Up of the Experimental Environment

**Figure 11.**
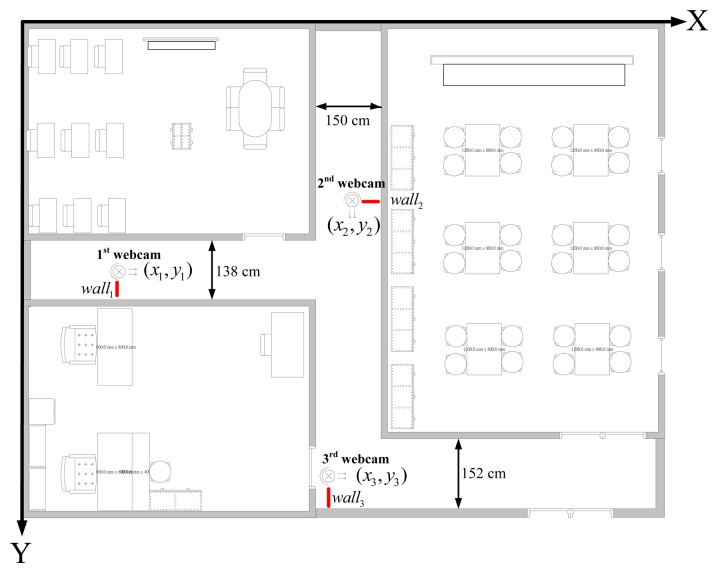
Map of our experimental environment.

Figure 11 shows the map of our experimental environment. In this map three webcams, which are explained in Section 2.1, are used to cover the possible working area of the robot. The coordinates (*x*_1_; *y*_1_) of the first webcam are (279; 570); (*x*_2_; *y*_2_) are (755; 465); and (*x*_3_; *y*_3_) are (650; 1,030). Because of limitation of the USB 2.0 transmission speed, the resolution of the first webcam is 640 × 480; the second webcam is 640 × 480; and the third webcam is 480 × 360.The aisle widths are shown in Figure 11, and are 138, 150 and 152 cm, respectively. The background image is updated in every 5 s. Threshold value *t*_1_ of the updated background image is set to 19.5.

### 4.2. IBDMS and PLDMS Set-Up

**Figure 12.**
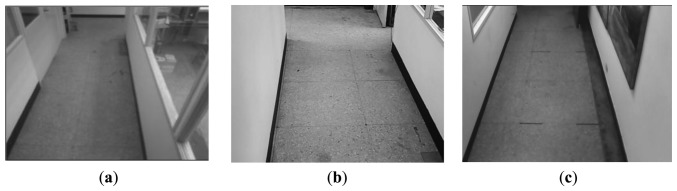
(**a**) Background image of the 1st webcam. (**b**) Background image of the 2nd webcam. (**c**) Background image of the 3rdwebcam.

Some basic set-up steps must be performed for the IBDMS and PLDMS before we start the robot localization procedures. In order to alleviate the impact from the image noise, building the first background image is done taking the average of 150 consecutive images, and then the background subtraction method can much more effectively extract the foreground image. Besides, w low-pass filter is adopted to further refine the background image. The obtained background images are shown in Figure 12.

**Figure 13.**
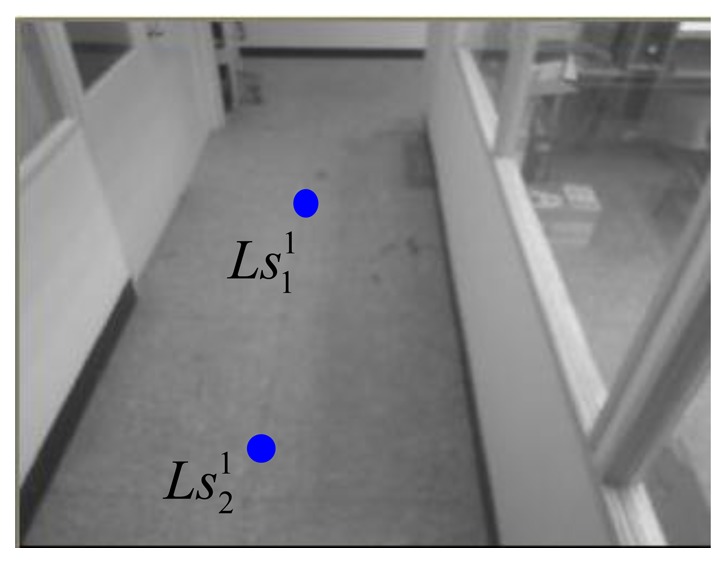
Two chosen points for the right line in the 1st webcam.

**Figure 14.**
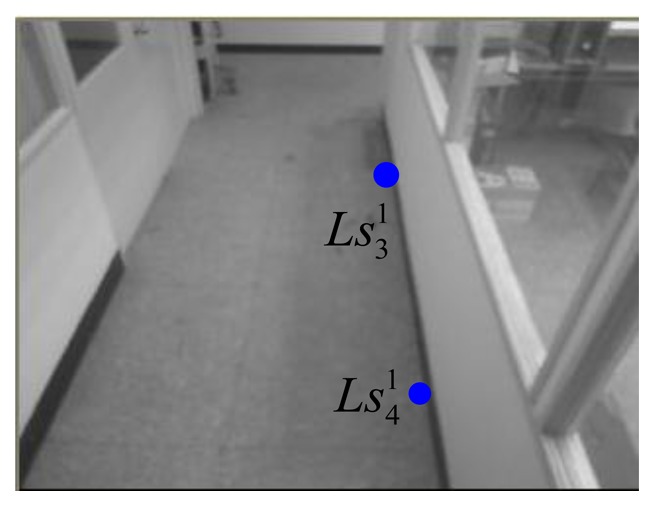
Two chosen points for the left line in the 1st webcam.

In the 1st webcam, as shown in Figures 13 and 14, four corners of the known-dimension ground tile are used to draw a pair of the virtual parallel line ( $\bar{Ls_{1}^{1}Ls_{2}^{1}}$ and $\bar{Ls_{3}^{1}Ls_{4}^{1}}$), whose deriving linear equations can be expressed as:

(24) $$\bar{Ls_{1}^{1}Ls_{2}^{1}}:y_{1}^{1}=-6.0625x_{1}^{1}+865.063$$

and:

(25) $$\bar{Ls_{3}^{1}Ls_{4}^{1}}:y_{2}^{1}=5.68x_{2}^{1}-992$$

where the coordinates of $Ls_{1}^{1}$, $Ls_{2}^{1}$, $Ls_{3}^{1}$, and $Ls_{4}^{1}$ are respectively chosen as (113, 180), (129, 83), (112, 181), and (128, 97) in the image-domain.

**Figure 15.**
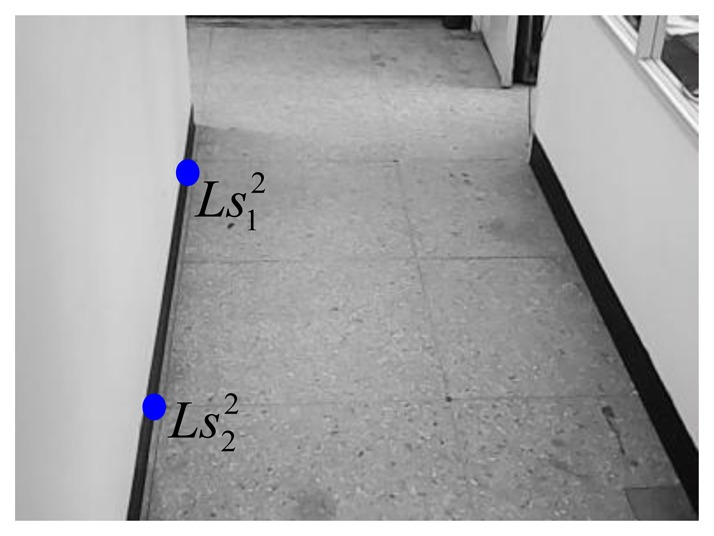
Two chosen points for the right line in the 2nd webcam.

**Figure 16.**
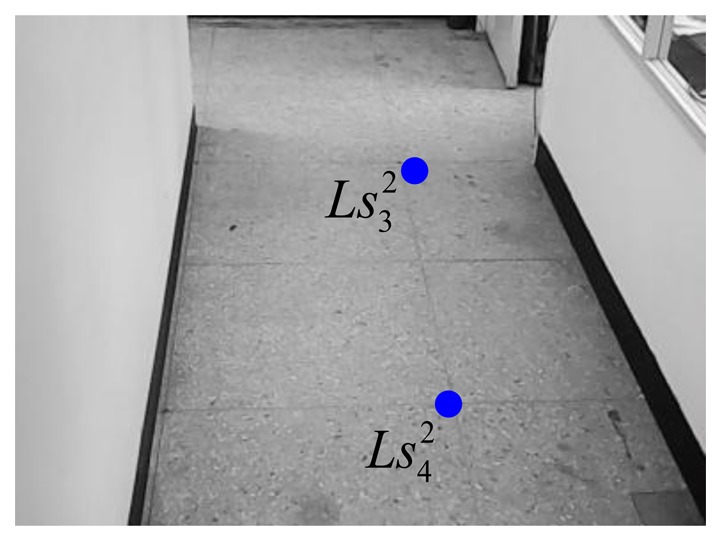
Two chosen points for the left line in the 2nd webcam.

Similar to the setting procedures of the 1st webcam, the virtual parallel line ( $\bar{Ls_{1}^{2}Ls_{2}^{2}}$ and $\bar{Ls_{3}^{2}Ls_{4}^{2}}$) for the 2nd webcam can be expressed as:

(26) $$\bar{Ls_{1}^{2}Ls_{2}^{2}}:y_{1}^{2}=-8.107x_{1}^{2}+1500$$

and:

(27) $$\bar{Ls_{3}^{2}Ls_{4}^{2}}:y_{2}^{2}=4.551x_{2}^{2}-1479.61$$

where the coordinates of $Ls_{1}^{2}$, $Ls_{2}^{2}$, $Ls_{3}^{2}$, and $Ls_{4}^{2}$ are respectively chosen as (140, 365), (168, 138), (404, 359), and (355, 136) in the image-domain. Figures 15 and 16 show these four points.

**Figure 17.**
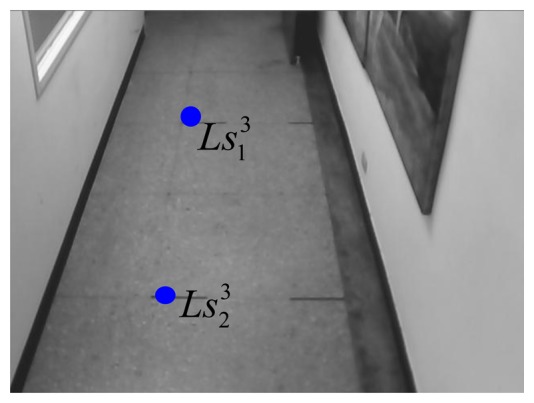
Two chosen points for the right line in the 3rd webcam.

**Figure 18.**
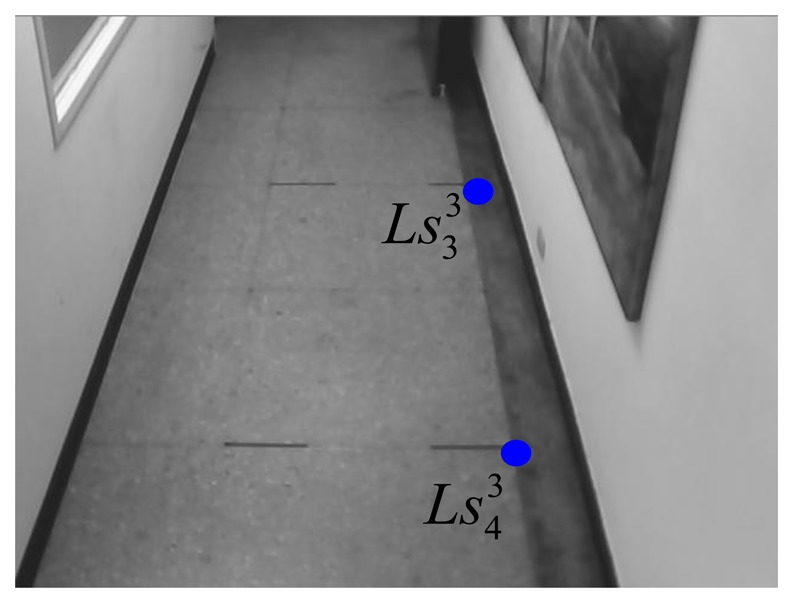
Two chosen points for the left line in the 3rd webcam.

The virtual parallel line ( $\bar{Ls_{1}^{3}Ls_{2}^{3}}$ and $\bar{Ls_{3}^{3}Ls_{4}^{3}}$) for the 3rd webcam can be expressed as:

(28) $$\bar{Ls_{1}^{3}Ls_{2}^{3}}:y_{1}^{3}=-5.475x_{1}^{3}+1309.65$$

and:

(29) $$\bar{Ls_{3}^{3}Ls_{4}^{3}}:y_{2}^{3}=5.815x_{2}^{3}-2055.55$$

where the coordinates of $Ls_{1}^{3}$, $Ls_{2}^{3}$, $Ls_{3}^{3}$, and $Ls_{4}^{3}$ are respectively chosen as (174, 357), (214, 138), (415, 358), and (377, 137) in the image-domain. Figures 17 and 18 show these four points.

### 4.3. Experimental Results

**Figure 19.**
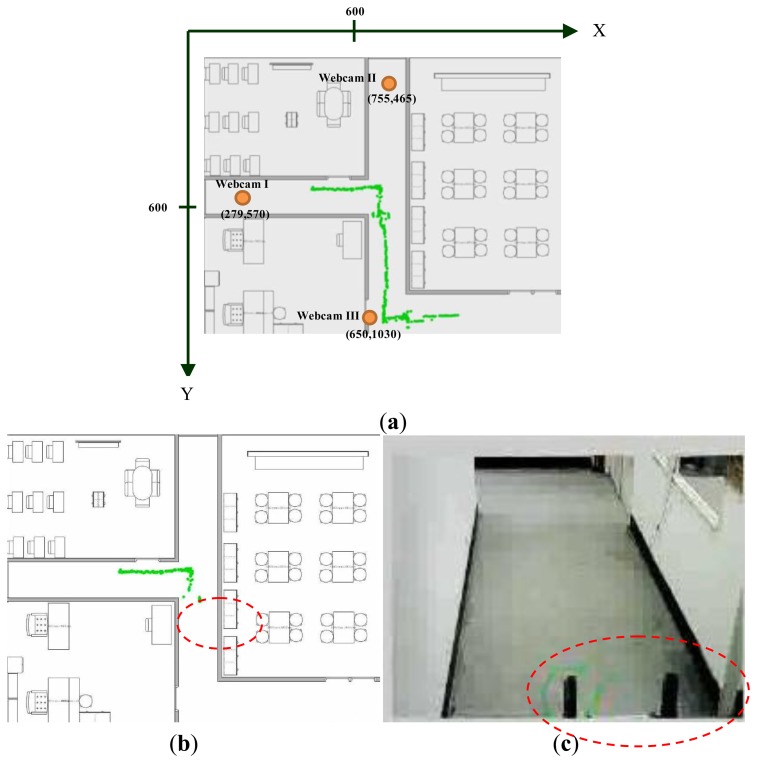

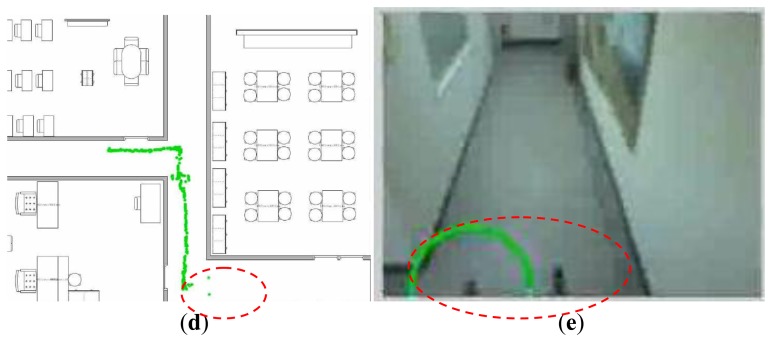
Moving path of the track robot (**a**) Path of the robot; (**b**) Path captured under the Webcam II (**c**) Image of the Webcam II; (**d**) Path captured under the Webcam III; (**e**) Image of the Webcam III

In our experiments, a remote-controlled track-robot moves through the monitored areas. The path of the robot is shown in Figure 19a. In Figure 19a, the circled locations, causing bigger errors as shown in Figure 19b, are the coordinates of the front arms of the robot at the moment of which the robot is moving into the covering area of the 2nd webcam, as shown in Figure 19c. In Figure 19d, a bigger error happens in the circled locations, which are the coordinates of the front arms of the mobile robot at the moment of which the robot is moving into the covering area of the 3rd webcam, as shown in Figure 19e. The error function used to show the ability of our proposed localization method is:

(30) $$\text{Error}=\left|\sqrt{{\left(x_{2}-x_{1}\right)}^{2}+{\left(y_{2}-y_{1}\right)}^{2}}\right|$$

where (*x*_1_, *y*_1_) are the actual coordinates, and (*x*_2_, *y*_2_) are the coordinates measured by the proposed method. In Table 1, the measurement errors range from 2.24 cm (when the robot is near the webcams) to 12.37 cm (when the robot is far away from the webcam). According to the definition of *R_center_* and the dimensions of the robot, which are 54 cm × 54 cm, a common size for patrolling robots, we find that the robot can be correctly located even though it is far away from the webcams. Limited by the low-resolution webcams, the measurement errors are acceptable. We also can easily reduce the measurement errors by using high-resolution CCD camera. Furthermore, the selection of a known-dimension rectangle pattern should be clearly seen in the captured image in order to set up the reference points of IBDMS and PLDMS. In addition, a suitable threshold value *t*_1_ in the segmentation horizontal projection, which is used to update the background image, and the distortion coefficient vectors and camera conversion matrix in image calibration are important factors for precisely locating the mobile robots.

Under the conditions of the static monitored area, it is assumed that light sources and locations of walls and furniture are given. Light influence, therefore, can be easily attenuated through choosing appropriate factors in the image processing techniques. In this paper, we pay more attention to locating the moving robot by using single webcam and have not yet considered the situation of partial occlusions. In this static indoor environment, some image techniques [33–35] could be used to overcome temporary partial occlusion.

## 5. Conclusions

This paper proposes the use of IBDMS and PLDMS to locate a mobile robot in an indoor environment. Through the image processing and according to a known-dimension ground tile, the IBMDS and PLDMS used can calculate the coordinates of a moving tracked robot. Using this framework, we can quickly estimate the localization of the tracked robot. Furthermore, the experimental environment is easy to set up since only three parameters have to be defined, that is, the maximum pixel, the perspective, and the optical distance. Because the locations of webcams are fixed, we can utilize a simple background subtraction method to extract the data to attenuate the problem of computational burden. In addition, we use a low-pass filter and an on-line background updating method to reduce background noise, and we adopt the image morphology to acquire the robot's image information. This method does not use expensive high-resolution webcams and complex pattern recognition methods to identify the mobile robot, but rather just uses a simple formula to estimate distance. From the experimental results, the localization method is both reliable and effective.

## Figures and Tables

**Table 1.** Measurement errors of the proposed method.

**Actual Coordinate**	**Measured Coordinate**	**Error (cm)**	**Webcams**
(610,592)	(613,588)	5.00	Webcam I
(627,611)	(626,609)	2.24	Webcam I
(636,560)	(630,559)	8.08	Webcam I
(648,591)	(641,594)	7.62	Webcam I
(684,584)	(676,578)	10.00	Webcam I
(736,891)	(738,883)	8.24	Webcam II
(774,912)	(775,901)	11.04	**Webcam II**
(759,918)	(756,906)	12.37	Webcam II
(763,944)	(759,932)	12.65	Webcam II
(808,966)	(811,952)	12.37	Webcam II
(1001,779)	(997,779)	4.00	Webcam III
(1236,1076)	(1225,1074)	11.18	Webcam III
(1250,1019)	(1241,1021)	9.49	Webcam III
(1271,1063)	(1262,1065)	11.18	Webcam III
(1250,1041)	(1241,1039)	9.22	Webcam III
